# The Role of Purinergic P2X7 Receptor in Inflammation and Cancer: Novel Molecular Insights and Clinical Applications

**DOI:** 10.3390/cancers14051116

**Published:** 2022-02-22

**Authors:** John Charles Rotondo, Chiara Mazziotta, Carmen Lanzillotti, Chiara Stefani, Giada Badiale, Giulia Campione, Fernanda Martini, Mauro Tognon

**Affiliations:** 1 Laboratories of Cell Biology and Molecular Genetics, Section of Experimental Medicine, Department of Medical Sciences, School of Medicine, University of Ferrara, 44121 Ferrara, Italy; rtnjnc@unife.it (J.C.R.); chiara.mazziotta@unife.it (C.M.); carmen.lanzillotti@unife.it (C.L.); chiara.stefani@univr.it (C.S.); giada.badiale@unife.it (G.B.); giulia01.campione@edu.unife.it (G.C.); mrf@unife.it (F.M.); 2 Centre for Studies on Gender Medicine, Department of Medical Sciences, University of Ferrara, 44121 Ferrara, Italy; 3 Laboratory for Technologies of Advanced Therapies (LTTA), University of Ferrara, 44121 Ferrara, Italy

**Keywords:** P2X7 receptor, P2X7R, ATP, BzATP, tumour microenvironment, inflammation, immune system, colon cancer, melanoma, infection, malignant pleural mesothelioma

## Abstract

**Simple Summary:**

The purinergic P2X7 receptor (P2X7R) is a cell membrane protein whose activation has been related to a variety of cellular processes, while its dysregulation has been linked to inflammation and cancer. ATP plays a key role in numerous metabolic processes due to its abundance in the tumour microenvironment. P2X7R plays an important role in cancer by interacting with ATP. The unusual property of P2X7R is that stimulation with low ATP doses causes the opening of a permeable channel for sodium, potassium, and calcium ions, whereas continued stimulation with high ATP doses leads to the formation of a non-selective pore. The latter effect induces the cell death. This evidence suggests that P2X7R has both pro- and anti-tumour potential. In this review, we aimed to describe the most relevant characteristics of P2X7R function, activation, and its ligands, while also summarising the role of P2X7R activation in the context of inflammation and cancer. The currently used therapeutic approaches and clinical trials of P2X7R modulators is also described.

**Abstract:**

The purinergic P2X7 receptor (P2X7R) is a transmembrane protein whose expression has been related to a variety of cellular processes, while its dysregulation has been linked to inflammation and cancer. P2X7R is expressed in cancer and immune system cell surfaces. ATP plays a key role in numerous metabolic processes due to its abundance in the tumour microenvironment. P2X7R plays an important role in cancer by interacting with ATP. The unusual property of P2X7R is that stimulation with low doses of ATP causes the opening of a permeable channel for sodium, potassium, and calcium ions, whereas sustained stimulation with high doses of ATP favours the formation of a non-selective pore. The latter effect induces a change in intracellular homeostasis that leads to cell death. This evidence suggests that P2X7R has both pro- and anti-tumour proprieties. P2X7R is increasingly recognised as a regulator of inflammation. In this review, we aimed to describe the most relevant characteristics of P2X7R function, activation, and its ligands, while also summarising the role of P2X7R activation in the context of inflammation and cancer. The currently used therapeutic approaches and clinical trials of P2X7R modulators are also described.

## 1. Background

**Figure 1:**
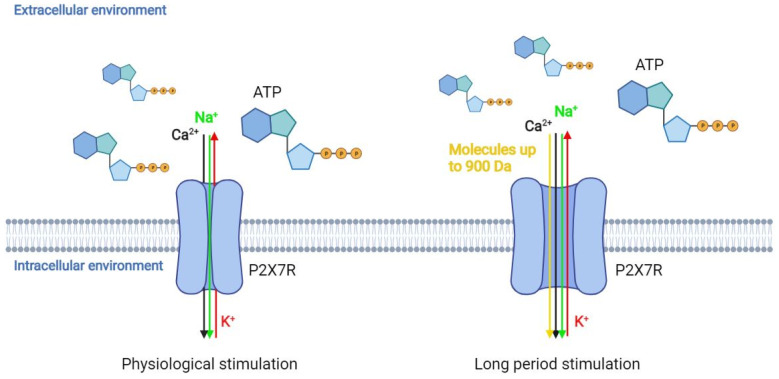
Schematic representation of P2X7 receptor (P2X7R). P2X7R is an ion channel permeable to Na^+^, K^+^, and Ca^+2^. P2X7R physiological stimulation with ATP leads to channel opening for Na^+^, K^+^, and Ca^+2^, while long-term stimulation of P2X7R with ATP causes the opening of a non-selective pore that allows the passage of molecules whose molecular weight is lower than 900 Da.

Extracellular nucleotides, including adenosine triphosphate (ATP), play a fundamental role in various biological functions, such as cell growth, differentiation, and migration, as well as tissue homeostasis, immunity, inflammation, and cancer [1]. These molecules act as messengers in cell-to-cell communication by stimulating the release of other extracellular factors, which in turn activate additional signalling pathways by interacting with their own receptors [2]. One of the main functions of these molecules is the modulation of purinergic signal transduction [2]. ATP is found at high concentrations (5–10 mM) in physiological conditions [3]. It interacts with immune cells, the vascular endothelium, and the surrounding matrix. When ATP is transferred from the cell cytoplasm to the extracellular environment, it can act as a damage-associated molecular pattern (DAMP), i.e., it acts as a signal molecule that induces an inflammatory response [4]. Consistently, extracellular ATP (eATP) accumulates near the inflamed tissues [4]. Moreover, both adenosine and ATP act as regulators in tumour growth processes. Both molecules are abundant in the tumour microenvironment (TME), comprising tumour-surrounding blood vessels, immune cells, fibroblasts, signalling molecules, and the extracellular matrix [5]. A key ATP target is represented by the purinergic P2X receptor (P2XR) family members [6]. These receptors are trimeric ATP-activated ion channels that are permeable to Na^+^, K^+^, and Ca^+2^, which open when interacting with eATP [7]. The opening of non-selective pores causes the dysregulation of cellular homeostasis with consequent cell death [8]. However, P2XR activation causes the release of cytokines, guides survival, metabolic adaptation to nutrient deprivation, and migration and invasion of cancer cells [9]. Particular attention has been paid to the P2X7 receptor (P2X7R) [10], whose expression is associated with inflammation, survival, proliferation, angiogenesis, and metastasis [11,12]. This receptor, which is found on both cancer and immune system cell surfaces, is characterised by a biphasic response to ATP: (i) a short stimulation allows the influx of sodium and calcium ions into the cell; (ii) a long period of stimulation of the receptor triggers the opening of a non-selective pore that allows the passage of molecules whose molecular weight is lower than 900 Da (Figure 1).

In low ATP concentration conditions in the TME, ranging from 0.1 to 0.5 mM, P2X7R activation promotes tumour growth due to limited entry of Ca^+2^ through the channel [13]. Contrariwise, high ATP concentrations cause P2X7R activation that induces cell death as high Ca^+2^ input through the pore becomes toxic [14,15]. P2X7R and its ligands play a key role in tumorigenesis, development, and metastasis in a variety of cancers. Its role in inflammation is not fully understood.

In this review, we aimed to describe P2X7R activation and functions, and the effects of P2X7R interaction with its ligands on cancer and immune response, as well as its role in inflammation and tumour-associated inflammation. We also describe the currently employed therapeutic approaches and the clinical trials of P2X7R modulators.

## 2. P2X7 Receptor Genetics, Characteristics, Function, and Tissue Distribution

The P2XR family includes seven different subtypes, i.e., P2X1-7, which are typed according to their pharmacological properties and structures. These receptors are cation-permeable ligand-gated ion channels that open in response to the binding of eATP consisting of three protein subunits (homotrimers or heterotrimers) activated by ATP permeable to Na^+^, K^+^, and Ca^+2^. The P2XR coding genes map in different chromosome loci, whilst homologous genes have also been identified in other vertebrates [16]. The *P2X7R* (or *P2RX7*) gene maps in the 12q24.31 chromosome [17], containing 13 exons and 10 splicing variants (P2X7A-J) [18], while the full-length isoform is the P2X7A form. Evidence indicates that P2X7R expression appears to be epigenetically regulated by DNA methylation [19] and miRNA regulation [20], which are important processes for gene expression regulation in a variety of cell types [21,22,23,24,25]. The P2X7R full-length isoform consists of a sequence of 595 amino acid (a.a.) residues, in which two hydrophobic regions crossing the plasma membrane can be identified: (i) N-terminus (N-ter), which is located in the cytoplasm; (ii) C-terminus (C-ter), which contains 70 to 200 additional a.a. residues compared to other P2X family receptors [26,27]. The extracellular domain contains the ATP binding site and 10 cysteine residues whose oxidation contributes to the sulphide bridge formation, which is necessary for the tertiary structure [26]. The C-ter domain is involved in the majority of functions related to P2X7R, such as post-translational modifications (PTMs), cell localisation, protein–protein interaction, and initiation of the signal transduction cascade [26]. PTMs occurring at this domain play a role in the receptor function through regulating its signalling pathways. For instance, a tyrosine phosphorylation site within the C-ter domain has been related to the P2X7R-induced release of TNF-α [28]. The cysteine residues located at the P2X7R domain are susceptible to palmitoylation, which is a PTM capable in influencing both receptor function and trafficking to and/or from the cell surface membrane [29]; P2X7R C-ter palmitoylation in particular facilitates the receptor large pore opening and enhances the association of the C-ter a.a. residues to the plasma membrane cholesterol [30]. Mutations at this domain can reduce both cation channel activity and pore formation [31], while also influencing, at the same time, P2X7R trafficking [32]. Likewise, mutations at the C-ter domain can result in a decreased receptor function through defective N-linked glycosylation processing and oligomerisation [31,33,34,35]. P2X7R C-ter domain is involved in the interaction between the receptor and membrane structural factors and/or intracellular signalling messengers in order to mediate signalling transduction pathway activation [32,36]. For instance, the P2X7R C-ter domain is capable of interacting with the adaptor protein of the NF-κB, i.e., MyD88, in order to provoke NF-κB activation, ultimately leading to the positive regulation of cytokine expression such as immature pro-interleukin-1β (pro-IL-1β) [37]. Moreover, the cysteine-rich domain located within the C-ter of the receptor prevents receptor desensitisation [38] while contributing to Ca^2+^-independent facilitation current [36]. Different proteins interacting with P2X7R C-ter domain have been identified with different assays [39], including cytoskeletal proteins and enzymes such as kinases, intracellular/transmembrane heat shock proteins other than the P2X4 receptor (P2X7R), and pannexin-1 plasma membrane hemichannels. The latter in particular mediates the P2X7-dependent pore opening as well as IL-1β and ATP release [40,41].

Crystallographic studies have shown that P2X7R consists of three subunits, which include the ATP binding site in the pocket structure [38]. The ATP binding site has seven positively charged a.a. residues and two hydrophobic a.a. residues. There are four lysine residues (Lys64, Lys66, Lys193, and Lys311) that are responsible for the inhibition of the receptor following binding with oxidised ATP (oATP). P2X7R sequencing/structural analysis suggested that the ATP binding site allows small molecule access [42]. The receptor also presents an allosteric site in the interface between the subunits located adjacent to the ATP binding site [38]. When the allosteric site is occupied, a conformational change in the receptor is induced, leading to the channel opening. This site is absent in P2X3-4 receptors [42]. P2RX7 has a consensus sequence (Ans-X-Ser/Thr) being located within the three glycosylation sites of the protein. Glycosylation is a post-translational modification, essential for the molecular transport of the receptor on the cell surface and for the receptor function. If two-thirds of sites are glycosylated, the receptor is fully functional. If only one site is glycosylated, the response to ATP is barely detectable, while in conditions of no glycosylation, the receptor does not respond to stimulation [43]. This characteristic is shared with all P2X family receptors [43]. The human P2X7R is a highly polymorphic gene. Several single-nucleotide polymorphisms (SNPs) have been identified within the P2X7R coding gene. Most SNPs have been found as being located within the intronic regions, while about 150 non-synonymous SNPs have also been identified [26]. Mutations can be neutral or can positively or negatively affect P2X7R activity. The combination of these SNPs generates different haplotypes with effects on the receptor functionality. Thus, it is impossible to predict receptor functionality by identifying only one SNP. The most common SNPs and related diseases/conditions, including inflammation-related diseases and cancer, are listed in Table 1 [42].

The role of SNPs within the *P2X7R* coding gene is not yet completely clear. However, it is known that in the case of loss-of-function SNPs, the receptor may promote cell growth by inhibiting apoptosis, while in the case of gain-of-function SNPs, P2X7R induces cell proliferation and the release of factors, such as vascular endothelial growth factor (VEGF) and transforming growth factor beta (TGF-β) [44]. In addition, only a few gene mutations clearly interfere with receptor functionality [45]. Indeed, not only is P2X7R highly expressed in cancer, but tumour tissues express the functional form of the receptor [46,47]. The unusual characteristic of P2X7R is that stimulation with low doses of ATP causes the opening of a permeable channel to Na^+^, K^+^, and Ca^+2^ ions [48], but continuous stimulation at high doses of ATP leads to the formation of a non-selective pore, which is permeable to molecules with a molecular weight of up to 900 Da (Figure 1) [49,50]. The mechanism responsible for the channel-to-pore transition is not completely clear. It has been hypothesised that continuous stimulation with ATP recruits accessory proteins involved in pore formation [51]. Another hypothesis is that the trimer can change its conformation, enlarging the diameter of the channel [52]. Indeed, previous data suggest that the non-selective pore formation is due to both the recruitment of accessory molecules and the dilation of the channel itself. Thus, both hypotheses could be considered valid. A possible explanation could be that the single binding between ATP molecule and P2X7R and the subsequent conformational change of the binding domain makes the second binding with another ATP molecule more difficult. When a second ATP molecule binds to P2X7R, the channel permeable to cations opens alongside another conformational change, which increases the difficulty of binding a third ATP molecule. However, binding with the third ATP molecule is still possible. When this interaction occurs, P2X7R undergoes another conformational change, thus causing the dilation of the channel, which becomes permeable to molecules with molecular weight of up to 900 Da. In this condition, there is an increase in the flow of cations into the cell [53]. This process activates other pores, which are responsible for the transport of large molecules. An additional characteristic of P2X7R is that the receptor is not desensitised in the presence of the agonist, even after several minutes [26]. Sustained agonist stimulation increases receptor sensitivity and the amplitude of the response. Contrariwise, P2X1/-3 receptors are desensitised in a few seconds, while P2X2/-4/-5 receptors are desensitised after less than a minute [26]. Cell membrane composition affects the pore opening. For instance, high cholesterol content in the membrane inhibits pore openings [42]. Indeed, studies aimed at identifying the mechanism of cholesterol action have suggested that the inhibition of pore openings does not depend on changes in membrane fluidity; rather, it is likely that cholesterol directly interacts with the P2X7R trans-membrane domain [42]. P2X7R is expressed in hematopoietic cells, such as monocytes, macrophages, dendritic cells, B and T lymphocytes, and Langerhans epidermal cells, but also in the basolateral membrane cells in the liver, as well as in osteoclasts and osteoblasts [53,54,55,56]. P2X7R overexpression/activation is involved in many different pathological processes, such as inflammation [57], proliferation, invasion and migration, metabolism, autophagy, and cell death [53,58,59]. Receptor activation can either present positive or negative effects in relation to the duration and intensity of the stimulation, the cell type, the ion extracellular concentration, and the phospholipid composition of the membrane plasma [26,46,53,60].

## 3. The Role of P2X7 Receptor in Cancer

A crucial P2X7R characteristic is that prolonged receptor stimulation leads to the opening of a non-selective pore, allowing for the passage of several molecules with molecular weights of up to 900 Da. This event causes an alteration in the intracellular homeostasis, which can lead to cell death. However, as it has been reported that P2X7R activation confers an advantage for cancer cell survival and growth, the role of the receptor in cancer is enigmatic [61]. Moreover, when the cell type is considered, the effect of receptor activation is different, and, in some cases, even contrary, thus indicating that the receptor presents both pro- and anti-tumour activities [26]. However, a tumour diagnostic/prognostic role for P2X7R has been remarked upon [62,63], as it has been found that P2X7R is overexpressed in a variety of cancers, such as lung, colon, thyroid, pancreatic, prostate, and breast cancer, as well as lymphoma and glioma [8,64].

Overexpression of P2X7R leads to an increase in tumour growth, metastasis, production of VEGF, and release of matrix metalloproteinases (MMPs) [65], which are proteolytic enzymes that degrade extracellular matrix proteins and play a key role in metastasis [47]. Tumours expressing P2X7R are also characterised by increased proliferation, decreased apoptosis, and high levels of the transcription factor nuclear factor of activated T cells 1 (NFATc1). P2X7R-related tumours also show alteration of intracellular Ca^2+^ homeostasis and stimulation of mitochondrial metabolism [66]. At the same time, NF-κB accumulates in the nucleus and contributes to cell invasiveness and survival by maintaining high P2X7R expression and inducing synthesis of MMP-3 and MMP-9 [67]. Under hypoxic conditions, the expression of P2X7R increases. Knockdown of hypoxia-inducible factor 1α (HIF-1α) decreases P2X7R expression, which also decreases nuclear factor kappa light chain enhancer of activated B cells (NF-κB), thus suggesting that HIF-1α controls P2X7R and NF-kB expression under hypoxia conditions.

P2X7R induces apoptosis by activating caspases 3 and 7 following a massive Ca^+2^ intake [68,69]. At low ATP concentration, ranging from 0.1 to 0.5 mM, the receptor promotes tumour growth and metastasis formation, whilst strong P2X7R activation leads to cell death due to the cytotoxic effect of excessive Ca^+2^ influx [8]. Activation of P2X7R in melanoma, colon cancer, and neuroblastoma cancer cell lines induces secretion of VEGF [14,70,71], while its activation in breast cancer cell lines and leukocytes leads to an increase in the activity of MMP-3, MMP-9, and MMP-13 [14,72,73,74]. Specifically, MMP-9 plays an important role in intravasation, i.e., invasion of tumour cells through the basement membrane into a blood or lymphatic vessel [75]. One mechanism by which P2X7R regulates MMP activity is the release of cathepsin B, an enzyme that inactivates metalloprotease inhibitors [76]. P2X7R also induces intravasation by promoting epithelial–mesenchymal transition (EMT) [77]. Indeed, activation of the receptor after binding to MMP-3 leads to the loss of E-cadherin, a molecule that promotes cell adhesion. It has also been suggested that P2X7R may play a role in actin remodelling, a mechanism associated with EMT [14].

Lung cancer animal models indicate that P2X7R inhibition leads to a dramatic reduction in the migration of transplanted tumour cells into immunocompromised mice [78,79]. Pharmacological receptor blockade decreases the invasiveness of breast cancer cells [73,80], while ATP induces increased cell migration and metastasis by increasing the release of proteolytic enzymes that degrade the extracellular matrix, thereby promoting tumour invasiveness [14]. A study conducted with malignant pleural mesothelioma (MPM) cell lines reported that P2X7R stimulation induces cell proliferation even in the absence of cell nutrients [81]. This effect is not present in healthy mesothelial cells without P2X7R expression. Moreover, stimulation of P2X7R with agonists leads to an increase in intracellular Ca^+2^ in the absence of the pore. This is understandable, as despite P2X7R being expressed in MPM cells, the concentration of this receptor is insufficient in detecting the effects of pore opening. Another explanation might be that the cells may express a receptor isoform that does not favour pore opening [81].

In neuroblastoma, overexpression of P2X7R is involved in maintaining an undifferentiated cell state and decreases apoptosis. It also allows cell growth, even in the absence of serum or glucose [82]. Moreover, P2X7R is overexpressed in neuroblastoma cells and is associated with a negative prognosis [71]. Stimulation of P2X7R, and consequent activation of phosphoinositide 3-kinase (PI3K)/Akt/glycogen synthase kinase 3 beta (GSK-3β) [83], regulates aerobic glycolysis and cell cycle progression [71]. In particular, PI3K/Akt activation leads to an increase in GSK-3β phosphorylation, which is accompanied by a decrease in GSK-3β activity [84]. The non-phosphorylated form of GSK-3β mediates MYC proto-oncogene, bHLH transcription factor (*MYCN*) degradation. *MYCN* is an oncogene overexpressed in neuroblastoma [85]. PI3K activation is also associated with an increase in VEGF and HIF-1 in vivo and in vitro [71]. Moreover, P2X7R activation in neuroblastoma induces the release of substance P in a paracrine manner, which is associated with tumour growth [26].

The anti-proliferative/pro-apoptotic effect of activated P2X7R is associated with plasma membrane permeabilisation in several cancer types. However, in other cell types, the receptor functions as an ion channel but no pore formation occurs because a non-functional form of the receptor with a truncated C-ter is expressed. In this case, P2X7R controls other processes such as proliferation or invasiveness [26]. Another interesting effect of P2X7R stimulation is its ability to support and stimulate cell growth in the absence of serum [66].

## 4. P2X7 Receptor Activation in Inflammation

ATP and adenosine are present in modest amounts (nmol/L) in the interstitium of healthy tissues, while high levels of both molecules can be reached in the inflammatory microenvironment (IME), which is an essential component of the TME [86]. In physiological conditions, the concentration of eATP is in the nanomolar range, but in nearby inflamed sites, its concentration has been reported to be considerably higher. In addition, in TME, eATP concentrations can excess 700 µM [87]. The accumulation of ATP in IME and TME has several effects, including promotion of inflammatory cell migration; redirection of T helper cell differentiation; and activation of NLRP3 inflammasome, which is the most versatile and clinically significant inflammasome. The release promotion of chemokines, cytokines, and growth factors has also been reported. Additional effects comprise oxygen and nitrogen radical generation, stromal and/or tumour cell growth stimulation, potentiation of intracellular killing of pathogens, and direct cytotoxicity [88]. Furthermore, since P2X7R is upregulated on immune cells during inflammation process, the receptor can be considered as an immunomodulatory receptor [89]. ATP released from injured cells acts as a danger signal by targeting the upregulated P2X7R and enhancing immune responses involving the secretion of inflammatory cytokines, which occurs through the cell membrane by exocytosis [89,90,91]. Extracellular ATP is considered an endogenous adjuvant that can trigger inflammation by acting as a danger signal via stimulation of P2Rs. Among P2Rs, P2X7R is the most studied from an immunological point of view, being found to be involved in both innate and adaptive immune responses [88]. ATP acting through P2X7R is the second signal for inflammasome activation, inducing both the maturation and release of proinflammatory cytokines IL-1β and IL-18 and the production of oxygen and nitrogen radicals [88]. In addition, during the adaptive immune response, P2X7R modulates the balance between the generation of type 17 T helper lymphocytes (Th17) and regulatory T lymphocytes (Treg) [88].

Several diseases have been associated with P2X7R-mediated inflammation: diabetes gut, liver, kidney, respiratory tract, and cardiovascular diseases [88]. As a result, a large number of clinical trials based on the use of P2X7R inhibitors for the treatment of inflammation and related diseases have been developed. Indeed, since 1999, when AstraZeneca first reported the potential therapeutic application of P2X7R inhibitors for the treatment of inflammation-related diseases, several patents have been developed [92].

### 4.1. P2X7R in Pathogenic Infection-Driven Inflammation

The ATP release from immune and non-immune cells can increase as a response to pathogenic infections, while subsequent activation of P2X7R following ATP binding leads to the modulation of innate/adaptive immune responses [93,94]. For this reason, growing evidence indicates that P2X7R plays an important role in pathogen infection-driven inflammation [88,95,96]. The receptor has therefore been described as involved in the host response to various pathogenic infections including viruses, bacteria, fungi, protozoa, and even helminths [96].

ATP-P2X7R signalling modulates immune responses against several virus types. For instance, in vitro data indicated that P2X7R, when interacting with ATP, is able to protect marrow-derived macrophages from vesicular stomatitis virus (VSV) infection. The mechanism behind this process comprise the induction of IFN-β release via P38/JNK/ATF-2 signalling activation, which leads to the reduction of VSV replication levels [97]. Similar effects have also been demonstrated in vivo in VSV-infected WT mice, but not in P2X7R KO mice [97]. P2X7R activation plays a role in controlling infection of human monocytes by dengue virus-2 [98]. In addition, it seems that P2X7R favours an exacerbated immune response acting as a positive regulator of inflammation, according to the severity of the viral infection [99]. The purinergic pathway and in particular eATP-P2X7R pathway is implicated in human immunodeficiency virus (HIV) infection, in different cellular processes of the acquired immunodeficiency syndrome (AIDS) [100,101,102], and in hepatotropic virus infections such as hepatitis B virus (HBV) and hepatitis delta virus (HDV) [103]. In particular, hepatotropic viruses require the activity of P2X1 receptor (P2X1R), P2X4R, and P2X7R to infect primary human hepatocytes since the expression of these purinergic receptors in peripheral blood mononuclear cells (PBMCs) was found to be increased in chronic hepatitis C virus (HCV)-infected patients in comparison to healthy subjects [103]. A possible role of P2X7R signalling has been hypothesised in the etiopathogenesis of coronavirus disease (COVID-19) caused by severe acute respiratory syndrome coronavirus 2 (SARS-CoV-2) [104,105,106]. Indeed, P2X7R hyperactivation and consequent NLRP3 inflammasome activation has been found in response to SARS-CoV-2 infection [104]. For this reason, pharmacological P2X7R inhibition could be considered a helpful therapeutic approach for COVID-19 management.

An important antimicrobial activity through the production of inflammatory mediators in phagocytic cells and the modulation of the adaptive immune response against bacterial infections has been attributed to P2X7R [88]. The most studied pathogenic infections in the context of P2X7R pathway comprise (i) *Chlamydia trachomatis*, (ii) *Mycobacterium tuberculosis* and *bovis*, and (iii) Gram-positive bacteria. Regarding the bacterium, which is a common sexually transmitted pathogen [107], it has been reported that ATP-dependent P2X7R activation is capable of decreasing the bacterial DNA load in both epithelial cells and macrophages infected with several types of *Chlamydia* species [108] while boosting the anti-*Chlamydia* immune response via NLRP3 inflammasome activation and IL-1b release [109]. The inhibition of P2X7R signalling can also lead to antimycobacterial activities. For instance, P2X7R inhibition with Brilliant blue G (BBG) can prevent the development of a severe form of *Micobacterium tuberculosis*-driven tuberculosis in mice with experimental advanced pulmonary tuberculosis [110], while the adoptive transfer of P2X7R KO hematopoietic cells in mice can lead to a reduced pneumonia as well as a lower lung *Micobacterium bovis* burden in comparison with WT mice [95]. Furthermore, P2X7R has been reported to be implicated during the Gram-positive bacterial infection responsible for *sepsis*. Data obtained with an animal model of *sepsis* based on an ADORA2b KO mouse indicated that P2X7R on macrophages can reduce the bacterial DNA load and increase the release of inflammatory chemokines/cytokines while also improving mouse survival [111]. An additional study indicated that P2X7R inhibition with BBG can negatively modulate inflammation, cytokine production, and apoptosis of sinusoidal cells [112].

The implication of P2X7R signalling on pathogenic fungal infections has been described. For instance, P2X7R blockade can reduce the IL-1b levels in *Candida albicans*-stimulated PBMCs [113]. Data obtained from animal models indicated that P2X7R KO mice with pulmonary paracoccidioidomycosis, an infection caused by the fungus *Paracoccidioides*, presented reduced inflammation in comparison with WT mice [114]. In another study, *Alternaria alternata*-exposed mice under treatment with P2RX7 inhibitors showed an attenuated T helper 2 (TH2) response [115], which therefore reflected a reduction of the anti-parasite defence. Inversely, data obtained from a P2X7R KO mice model indicated the lack of a significant decrease in secretion of TH2 cytokines IL-5 and IL-13 [115].

The protective role of P2X7R for multiple protozoa, including *Plasmodium chabaudi*, *Leishmania amazonensis, Toxoplasma gondii,* and *Trypanosoma cruzi*, has been remarked upon. For instance, P2X7R overexpression/activation has been reported to be effective in counteracting several protozoal infections by (i) favouring NLRP3 inflammasome activation and stimulating IL-1b secretion, as occurs during *Toxoplasma gondii* infection [116] and (ii) mediating ATP-dependent host cell death, as demonstrated during *Leishmania amazonensis* infection [117].

Helminth is a group of large microparasitic worms that mainly infect the gastrointestinal tract and the blood vessels [118]. Current research has highlighted the protective role of P2X7R against helminth infection for the capacity of this purinergic receptor in boosting the anti-helminth host immunity [119]. During *Schistosoma mansoni* infection, the downregulation of P2X7R in mesenteric endothelial cells has been reported [118], while the simultaneous downregulation and overexpression of P2X7R and TGF-β, respectively, were determined in peritoneal macrophages [119]. Moreover, animal models conducted with P2X7R KO mice indicated a significantly higher fatality during *Schistosoma mansoni* infection compared to WT mice, who exhibited a complete survival [119]. It should be underlined that protozoa and helminths are eukaryotic organisms that both express P2X7R. As a consequence, modulators of P2X7R activity should be carefully investigated, being potentially effective against the parasitic PRX7R and that one expressed in the host [120,121].

In summary, these studies cumulatively indicate that the P2X7R signalling pathway plays a role in numerous aspects of pathogenic infection-driven inflammation, including immunomodulatory and proinflammatory activities. P2X7R can therefore be considered a possible therapeutic target for pathogenic infections management.

### 4.2. P2X7 Role in Tumour-Associated Inflammation

The TME comprises a large variety of immune cells, including monocytes, macrophages, dendritic cells, lymphocytes, and myeloid-derived suppressor cells, that mediate the induction of inflammatory processes and enable tumour growth and progression through direct interaction with cancer cells. This highly inflammatory milieu modulates the immune response against tumours, while P2X7R activation, expressed on dendritic cells, play a key role in this context by activating NLRP3 inflammasome [88]. NLRP3 have been shown to promote the onset/development of several cancers, including breast cancer, where it promotes the infiltration of myeloid cells, such as tumour-associated macrophages and myeloid-derived suppressor cells (MDSCs), which are key components of the immunosuppressive TME, favouring an IME and therefore promoting tumour initiation/progression [122]. The P2X7R-induced activation of NLRP3 also leads to IL-1β production and subsequent stimulation of CD4+ and CD8+ T lymphocytes, which mediate anti-tumour responses.

NLRP3 inflammasome activation also encompasses a functional interplay occurring between P2X7R, Pannexin-1 channel, and P2X4R [39,123]. These receptors/channels can interact with each other to form a membrane complex [124]; upon binding to ATP, the P2X4R/P2X7R/pannexin-1 complex can stimulate the production of active oxygen species (ROS), leading to the activation of NLRP3 inflammasome [124]. In vitro data obtained in breast cancer cells also indicated that the P2X4R/P2X7R/Pannexin-1 sensitivity to ATP can be modulated by a Food and Drug Administration (FDA)-approved anti-parasitic agent named ivermectin [125]. This interaction can prompt the channel opening, thus leading to the induction of mixed apoptotic and necrotic modalities of cell death alongside the activation of caspase-1 [125], which is a pro-inflammatory protein known to initiate pyroptotic cell death pathway activation [126]. These data cumulatively underline the importance of P2X4R/P2X7R/Pannexin-1 interplay upon inflammation and cancer, while opening a way for novel integrated cancer immunotherapy approaches.

The response following P2X7R activation not only affects cancer cells but also modulates the activity of cells with anti-tumour/immunosuppressive activity, or can stimulate the release of growth factors [44]. It seems that cancer cells benefit from receptor activation without, however, responding to the signal that should lead to cell death following the formation of the non-selective pore. This aspect is particularly intriguing, as the concentration of ATP is high in TME and therefore the formation of a pore would be expected.

Understanding the intracellular signalling pathways activated by P2X7R is still in the early stages. Numerous cell-specific signal transduction pathways are associated with receptor activation, such as PKC/MEK/ERK/FOS/JUN, PI3K/AKT/Mtor, MyD88/NF-κB, MMP-2/9, and calcineurin/NFATc1 [58,127]. Many of these signalling pathways are associated with the release of inflammatory mediators, such as caspase-1, IL-1β, IL-6, and NLRP3/ASC. Specifically, IL-1 β is a potent proinflammatory cytokine [128] whose ATP-induced release is regulated by alpha-1 antitrypsin (AAT) [129], a plasma protease inhibitor encoded by the *SERPINA1* gene [130,131]. By activating signal transduction pathways, P2X7R contributes to angiogenesis, invasion, and metastasis. Moreover, in vitro studies have shown that P2X7R expression causes cell infiltration and release of overexpressed extracellular matrix proteases in cancer cells [1,46]. P2X7R appears to inhibit tumour growth by promoting interaction between dendritic cells and tumour cells, pro-inflammatory cytokine release, chemotaxis, and infiltration of immune cells into the TME [1,71]. It also stimulates the release of immunosuppressive factors from MDSCs and modulates macrophages into the immunosuppressive phenotype M2, thus preventing the attack of natural killer and T cells on tumour cells [88].

High concentrations of ATP in the TME leads to an increase in the concentration of adenosine, which has immunosuppressive and anti-inflammatory effects [132]. Under these conditions, recruitment of immune system cells occurs, promoting anti-tumour immunity [132]. Indeed, T lymphocytes are activated against tumour cells by dendritic cells expressing P2X7R. Studies performed on various cancers, such as melanoma and colon cancer, show that tumour growth is accelerated in mice due to a reduced immune response. A functional P2X7R is required to activate the immune response [1,46]. The importance of P2X7R for an efficient anti-tumour response of immune system cells has been confirmed by bone marrow transplantation experiments from P2X7 WT mice to P2X7 KO mice, in which the anti-tumour response was restored [4]. Other data have reported that MDSCs overexpress P2X7R, thereby inducing the release of immunosuppressive factors, such as the potent anti-inflammatory cytokine IL-10 and TGF-β [133]. Therefore, treatment with antagonists acting on MDSC membrane receptors could restore an efficient antitumor response induced by the release of immunosuppressive factors [1]. P2X7R also plays a role in osteosarcoma progression and contributes to tumour-related pain perception [134]. In gliomas, P2X7R activation is associated with an increase in the expression of monocyte chemoattractant protein 1 (MCP-1), IL-8, and VEGF; increased inflammation; tumour cell migration; and caspase-3 and caspase-7 activation [44,135]. The role of the ectonucleotides CD39 and CD73, which hydrolyse ATP to adenosine, are interesting [136]. Adenosine has long been considered one of the main drivers of immunosuppression [137]. However, recent data show that P2X7R/CD39/CD73 axes also play important roles in modulating the TME immune component. P2X7R activation can be promoted by targeting CD39, which has a positive effect on antitumor responses and cannot be explained by inhibition of adenosine formation alone [138].

Additionally, a recent patent proposed a method of treating cancer by administering polymyxin B in combination with ATP as a novel tool to Treg CD4+ CD25+ lymphocytes, which prevent excessive immune responses and are known T-cell suppressors. In particular, the role of ATP in this patent is to stimulate P2X7R on Treg to promote apoptosis [139].

## 5. P2X7R as a Therapeutic Target in Cancer and Inflammation

### 5.1. P2X7R Agonists

P2X7R is differentially regulated depending on different ATP concentrations. At ATP concentrations above 3 mM, P2X7R can co-induce cell death. However, the receptor is also activated at low ATP concentrations, i.e., between 1 and 3 mM, which is typically found within the TME, especially near necrotic areas [140]. These low ATP concentrations are the reason for morphological changes leading to the acquisition of a pro-migratory phenotype, which is due to the activation of small conductance calcium-activated potassium channel 3 (SK3) following P2X7R stimulation and the consequent influx of calcium ions [74]. Moreover, P2X7R antagonist treatment has been shown to induce TGF-β1-induced reduction in cell migration in lung cancer cells [79]. In turn, TGF-β1 induces the release of ATP by activating the receptor in an autocrine/paracrine manner [79]. ATP inhibits the growth of prostate cancer cells, while activation of P2X7R in breast cancer cells induces apoptosis, thus negatively affecting cell proliferation [26]. Moreover, it has been reported that the anti-migratory effect induced by ATP is prevented under hypoxic conditions [141]. However, the identification of an alternative form of P2X7R, the so-called non-pore functional P2X7R (nfP2X7R) [142], whose stimulation is not associated with the opening of non-selective pores, complicates the situation [61]. It has been hypothesised that the high ATP concentration in the TME is responsible for the high nfP2X7R expression in cancer cells [61]. Indeed, experiments with myeloma cell lines have shown that incubation with ATP at a concentration between 0.1 and 0.25 mM overnight leads to a progressive decrease in pore function, which is completely abolished at a concentration of 0.5 mM [61]. These results indicate that cells are able to adapt to high ATP concentrations typical of TME after switching from the normal P2X7 form to nfP2X7. Incubation with ATP promotes a rapid downregulation of P2X7R, followed by a slow increase in the appearance of nfP2X7Rs on the surface [61]. In this way, cells benefit from the stimulation of nfP2X7Rs without cell death due to the opening of the pore [61]. It is still unclear as to which splice variant encodes the non-functional form of the receptor, but phase I clinical trials are ongoing to test the safety and tolerability of antibodies capable of recognising specific splice variants for the treatment of basal cell carcinoma [143].

The most potent P2X7R agonist is 2′,3′-benzoyl-4-benzoyl-ATP (BzATP) [144]. Although BzATP is an agonist, one that is 10–30 times more powerful than ATP, it is not selective for P2X7R as it also activates other P2XRs [144,145]. Both ADP and AMP are very weak P2X7R agonists, but prolonged ATP administration causes a conformational change, by which P2X7R loses the ability to distinguish among these three molecules [43]. Recent evidence from animal models indicated that NAD^+^ appears to be involved in P2X7R activation as CD38-mediated NAD^+^ degradation causes a decrease in receptor activation [42]. It is also conceivable that autocrine/paracrine NAD^+^ stimulates ATP secretion that, in turn, activates P2X7R [146]. Among the non-nucleotide agonists, β-amyloid peptide prompts responses normally associated with P2X7R stimulation in mouse microglial cells, which induces an influx of Ca^2+^, release of the pro-inflammatory cytokine IL-1β, and favours cytotoxicity [147]. P2X7R also seems to be activated by the bacterial peptide LL-37 [42], which induces the uptake of YO-Pro and the release of IL-1β in monocytes [148]. These effects are inhibited by administering receptor antagonists. The Polymyxin-β antibiotic acts as a positive allosteric modulator in HEK293 and K562 cells, which expresses P2X7R. This molecule increases the flow of Ca^2+^ ions, as well as the cytotoxic effect and the permeabilisation of the plasma membrane [149]. P2X7R also appears to be activated by liposaccharide, an endotoxin typical of Gram-negative bacteria [42].

### 5.2. P2X7R Antagonists

P2X7R blockers can be divided according to the P2X7R binding site. Some molecules share the same ATP binding site and therefore act as competitive antagonists, while other molecules bind other binding sites, thereby acting as allosteric modulators. P2X7R antagonists of the first generation include BBG and periodate-oxidised ATP. Although these compounds have been tested in several studies, they are not P2X7R-specific [150]. Indeed, they also inhibit P2X1 and -4 receptor activity [150]. Over the years, other antagonists that show greater selectivity for P2X7R have been developed, including AZ10606120 and A740003 [151,152]. Treatment with blockers is effective and well tolerated in different mouse models of inflammatory diseases, muscular dystrophy, and cardiomyopathy, as well as cancer [88,153]. Efficacy has also been proven in the treatment of psychiatric diseases such as Alzheimer’s and depression [154].

Different effects have been demonstrated on wild-type (WT) mice treated with P2X7R antagonists and P2X7R KO mice upon comparing the anti-tumour response of immune system cells [4]. In P2X7R KO mice, TME is characterised by a strong reduction in CD and Teff cells and an increase in Treg cells [4]. However, in the case of P2X7 WT mice, treatment with antagonists induces an increase in Teff cells but leaves the number of CD and Treg unaltered [4]. These data demonstrate that the use of antagonists has no side effects on the efficiency of the anti-tumour response in immune system cells that infiltrate the tumour. The effects of gene silencing and the pharmacological blockade of the receptor also have effects on the expression of ectoenzymes CD39 and CD73. These two ectoenzymes are involved in the formation of adenosine from ATP, which drives a shift from an ATP-induced proinflammatory milieu to an adenosine-driven anti-inflammatory environment [4]. The CD73 ectoenzyme has been found to be overexpressed in Treg cells infiltrating tumours in P2X7 KO mice, thus providing further evidence of the immunosuppressive role of Treg cells. CD73 is responsible for the hydrolysis of AMP into adenosine, which is known for its immunosuppressive and anti-inflammatory activity in TME [155]. Moreover, in P2X7 KO mice, there is an increase in CD73 and CD39 expression even in monocytes and Teff, which infiltrate the tumour. In case of P2X7 WT mice with implanted tumours, the use of antagonists, such as A740003, causes a downregulation of CD39 and CD73 in Teff and dendritic cells, suggesting that blocking the receptor may lead to a reduction in the degradation of ATP in adenosine [4], which triggers, in turn, an anti-inflammatory effect. In both melanoma and leukaemia cell lines, it has been seen that ATP levels are lower in mice with a silenced P2X7R coding gene [4]. This is due to on a defect in the release of ATP by cells from the immune system and an increase in ATP degradation mediated by infiltrating Treg cells. Treatment by AZ10606120 and/or A740003 in P2X7 WT mice causes an increase in the ATP released by the tumour cells, and therefore the use of these molecules can induce activation of an anti-tumour response [50]. Moreover, the use of antagonists does not cause an increase in the release of ATP by the immune cells that infiltrate the tumour [4]. The use of AZ10606120 and AZ740003 have also shown promising results in neuroblastoma and glioblastoma/glioma, respectively [71,156]. In neuroblastoma cells, P2X7R activation induces PI3K/Akt/GSK-3β/MYCN activation as well as HIF-1α/VEGF pathways. Additional molecules developed to inhibit PI3K/Akt activity have been shown to be effective in treating cancer.

P2X7R activation in melanoma, colon cancer, and neuroblastoma cells induces VEGF secretion [66]. A significant reduction in VEGF secretion and consequential reduction in the formation of new blood vessels was demonstrated in mice with melanoma treated with the antagonist AZ10606120 [71]. In a mouse model, the use of the periodate-oxidised adenosine 5′-triphosphate (oATP) antagonist induced blockage of tumour growth following a reduction in VEGF secretion. Similarity, an additional study found that implanting ACN cells in immunocompromised mice resulted in a significant reduction in VEGF [14]. An additional has been study conducted on several malignant pleural mesothelioma (MPM) cell lines immortalised with the T antigen (Tag) of simian virus (SV40), a small DNA tumour virus belonging to the polyomaviridae family [157,158,159]. It was shown that the effects of the pharmacological blockade of the receptor are variable according to the cell type examined. In SV40-Tag cells [160], receptor blockade with the selective agonist AZ10606120 or with the less specific antagonist oATP induces inhibition of cell proliferation and increases the release of lactate dehydrogenase (LHD), a common inflammation biomarker, associated with growth inhibition [81]. A similar result was also obtained in in vitro experiments in an additional study. BzATP showed cell growth-promoting activity. However, cell proliferation was fully blocked by oATP or by AZ10606120. To obtain more information on the role of the receptor in tumour growth, researchers conducted in vivo experiments by implanting MPM cells in *nude/nude* mice [81]. The P2X7R antagonist AZ10606120 was found to inhibit MPM growth, confirming the effects observed in vitro.

With the aim of developing novel MPM therapeutic approaches, researchers recently tested AZ10606120 in vitro in combination with an agonist of the adenosine A3 receptor (A3AR) [161]. The A3AR agonist Cl-IB-MECA and the P2X7R antagonist AZ10606120 were evaluated for their anti-tumour activities in vitro in MPM cells, including MPP89 and IST-Mes2, as a single agent or in combination [162]. Treatment with Cl-IB-MECA alone decreased cell proliferation and promoted apoptosis in both MPP89 and IST-Mes2, while AZ10606120 inhibited cell proliferation and induced apoptosis only in IST-Mes2 [162]. In addition, combined treatment with Cl-IB-MECA and AZ10606120 reduced cell proliferation and promoted apoptosis in MPP89 and IST-Mes2 cell lines, while no synergistic effect was detected [162].

The use of P2X7R antagonists induced the decrease in tumour size; the upregulation of the anti-oncogenic kinase GSK-3β; and the downregulation of MYCN, HIF-1α, and VEGF [71]. These data obtained from preclinical studies demonstrate the efficacy of P2X7R antagonists in the treatment of neuroblastoma [71]. In glioblastoma, P2X7R inhibition with AZ10606120 decreases granulocyte-macrophage colony-stimulating factor expression in U251 cells, thereby highlighting the potential therapeutic benefit of this P2X7R blocker [163]. Positive results on the efficacy of AZ10606120 have also been obtained using the PancTu-1 cell line in pancreatic cancer [164]. This model shows that migration and invasion is reduced in cells treated with AZ10606120. In addition, the use of the antagonist caused a reduction in the fibrous stroma associated with pancreatic cancer. Similar results have been obtained in other tumour types [81], thus validating the hypothesis that AZ10606120 has an anti-proliferative effect in vitro/in vivo.

Clinical trials have been designed to test the safety, tolerability, and clinical application of several P2X7R antagonists such as AZD9056, CE-224535, and GSK1482160 for the treatment of autoimmune and inflammatory diseases (www.clinicaltrials.gov, accessed on 1 February 2022). AZD9056 is a P2X7R inhibitor capable in counteracting the positive effect of BzATP on P2X7R activation [165]. The inhibitor has been tested for safety and efficacy in different phase I and II clinical trials for the treatment of rheumatoid arthritis (NCT00908934, NCT00700986, and NCT00520572), while an additional phase I clinical trial, which has been conducted in a cohort of healthy individuals, evaluated its pharmacokinetics in combination with simvastatin (NCT007366069), a compound used for reducing cholesterol/lipid levels [166]. The P2X7R inhibitor CE-224,535 has been repeatedly evaluated for the treatment of autoimmune diseases, including rheumatoid arthritis and osteoarthritis, in several phase I, II, and III clinical trials (NCT00418782, NCT00782600, NCT00446784, NCT00838058, and NCT00628095). Notably, the efficacy and safety of CE-224,535 has been tested without success in a phase II clinical trial (NCT00628095) for the treatment of patients with rheumatoid arthritis inadequately controlled by methotrexate, which is an immune system suppressant used for cancer and autoimmune diseases therapy [167]. Although the researchers hypothesised a possible anti-inflammatory effect of CE-224,535 via P2X7R inhibition and consequent IL-1 and IL-18 reduction, the presence of multiple pathways involved in the release of additional proinflammatory cytokines [128] may have sustained a chronic inflammatory status in these patients [168]. Regarding GSK1482160, a phase I clinical trial has been completed to test its efficacy and tolerability for treating inflammatory pain (NCT00849134). The use of GSK1482160 as a potential therapeutic agent for the treatment of cancer has also been hypothesised [169].

Notably, to the best of our knowledge, no clinical trials have been started to test the use of antagonists for the treatment of cancer since P2X7R activation induces an anti-tumour response in cells in the immune system that infiltrate the tumour and therefore the use of antagonists would seem to favour tumour progression. However, the data reported from preclinical studies are very promising, and therefore further tests could be conducted to validate the use of antagonists in the treatment of cancer. The antitumor effect of P2X7R antagonists has also been reported as being greater in immune-competent mice than in immune-deficient mice. This could also be due to the blocking of receptors expressed in MDSCs, in which activation of the receptor induces the release of immunosuppressive factors [71].

## 6. Conclusions and Future Perspectives

P2X7R is a purinergic receptor involved in inflammation and cancer. Its activation can have both pro- and antitumor effects, depending on the cell type and a variety of different factors. It is considered a key mediator of the antitumor immune response, but its expression on cancer cell membranes appears to mediate a dual role. It has been reported that P2X7R and its ligands play a central role in cancer development, progression, and metastasis. Previous in vitro studies and animal models have increasingly demonstrated the paramount importance of P2X7R in a variety of cancers, including lung, colon, thyroid, pancreatic, prostate, breast, lymphoma, MPM, and glioma. On the basis of these findings, P2X7R has recently emerged as an attractive potential diagnostic/prognostic marker and target for cancer therapy.

Clinical trials based on P2X7R antagonists are currently ongoing for the management of inflammatory diseases. In addition, although clinical trials have not yet been initiated to test the effect of P2X7R antagonists in the treatment of cancer, the preclinical data are very encouraging. In fact, a number of ligands have already shown promising results in animal models, while their clinical efficacy requires further investigation.

Investigating the role of P2X7R and its ligands in inflammation and in cancer development/progression is an important area of research. Since a considerable number of signalling pathways are involved in the stimulation of P2X7R, understanding the mechanism behind its activation is essential to deepen the role of this receptor in inflammation, tumour development, progression, and metastasis. In addition, the rationale for the dual nature of P2X7R in mediating cell proliferation and apoptosis under different pathophysiological conditions should be further investigated. The role of P2X7R in immune response is well established [170,171]. Therefore, targeting ATP/adenosine signalling may be a promising strategy to improve antitumor immunity in IME and TME. Further studies are needed to develop new strategies to block this signalling for the next generation of cancer immunotherapy. Therefore, further studies are needed to investigate the mechanisms of interaction between P2X7R and its ligands upon inflammation and cancer.

## Figures and Tables

**Table 1:** Main human P2X7 single-nucleotide polymorphisms and related diseases/conditions.

dbSNP ID	Base	Amino Acid	Effect on	Implicated Conditions
	Substitution	Substitution	P2X7 Function	
rsl7525809	370T > C	A76V	Loss	Multiple sclerosis
rs28360447	474G > A	G150R	Loss	Osteoporosis
rs208294	489C > T	H155Y	Gain	Multiple sclerosis, chronic pain, severe sepsis, children’s febrile seizures
rs7958311	835G > A	R270H	Loss	Chronic pain
rs28360457	946G > A	R307Q	Loss	Osteoporosis
rs1718119	1068G > A	A348T	Gain	Osteoporosis, anxiety disorder, toxoplasmosis
rs2230911	1096C > G	T357S	Loss	Osteoporosis
rs2230912	1405A > G	Q460R	Loss	Osteoporosis, severe sepsis, bipolar disorders, major depressive disorders
rs3751143	1513A > C	E496A	Loss	Osteoporosis, tuberculosis, cardiovascular risks
rs2230913	1563C > G	H521Q	Neutral	-
rs1653624	1729T > A	I568N	Loss	Osteoporosis

## References

[1] Di Virgilio F., Adinolfi E. (2017). Extracellular purines, purinergic receptors and tumor growth. Oncogene.

[2] Giuliani A.L., Sarti A.C., Di Virgilio F. (2019). Extracellular nucleotides and nucleosides as signalling molecules. Immunol. Lett..

[3] Greiner J.V., Glonek T. (2021). Intracellular ATP Concentration and Implication for Cellular Evolution. Biology.

[4] De Marchi E., Orioli E., Pegoraro A., Sangaletti S., Portararo P., Curti A., Colombo M.P., Di Virgilio F., Adinolfi E. (2019). The P2X7 receptor modulates immune cells infiltration, ectonucleotidases expression and extracellular ATP levels in the tumor microenvironment. Oncogene.

[5] Joyce J.A., Fearon D.T. (2015). T cell exclusion, immune privilege, and the tumor microenvironment. Science.

[6] Inami Y., Fukushima M., Kume T., Uta D. (2022). Histamine enhances ATP-induced itching and responsiveness to ATP in keratinocytes. J. Pharmacol. Sci..

[7] North R.A. (2016). P2X receptors. Philos. Trans. R. Soc. Lond. B Biol. Sci..

[8] Lara R., Adinolfi E., Harwood C.A., Philpott M., Barden J.A., Di Virgilio F., McNulty S. (2020). P2X7 in Cancer: From Molecular Mechanisms to Therapeutics. Front. Pharmacol..

[9] Burnstock G., Kennedy C. (2011). P2X Receptors in Health and Disease. Adv. Pharmacol..

[10] Xu X.Y., He X.T., Wang J., Li X., Xia Y., Tan Y.Z., Chen F.M. (2019). Role of the P2X7 receptor in inflammation-mediated changes in the osteogenesis of periodontal ligament stem cells. Cell Death Dis..

[11] Yang C., Shi S., Su Y., Tong J.S., Li L. (2020). P2X7R promotes angiogenesis and tumour-associated macrophage recruitment by regulating the NF-κB signalling pathway in colorectal cancer cells. J. Cell. Mol. Med..

[12] Adinolfi E., De Marchi E., Orioli E., Pegoraro A., Di Virgilio F. (2019). Role of the P2X7 receptor in tumor-associated inflammation. Curr. Opin. Pharmacol..

[13] Popper L.D., Batra S. (1993). Calcium mobilization and cell proliferation activated by extracellular ATP in human ovarian tumour cells. Cell Calcium.

[14] Hope J.M., Greenlee J.D., King M.R. (2018). Mechanosensitive Ion Channels: TRPV4 and P2X7 in Disseminating Cancer Cells. Cancer J..

[15] Notomi S., Hisatomi T., Kanemaru T., Takeda A., Ikeda Y., Enaida H., Kroemer G., Ishibashi T. (2011). Critical Involvement of Extracellular ATP Acting on P2RX7 Purinergic Receptors in Photoreceptor Cell Death. Am. J. Pathol..

[16] Hou Z., Cao J. (2016). Comparative study of the P2X gene family in animals and plants. Purinergic Signal..

[17] Burnstock G., Knight G.E. (2004). Cellular distribution and functions of P2 receptor subtypes in different systems. Int. Rev. Cytol..

[18] Benzaquen J., Heeke S., Janho dit Hreich S., Douguet L., Marquette C.H., Hofman P., Vouret-Craviari V. (2019). Alternative splicing of P2RX7 pre-messenger RNA in health and diseases: Myth or reality?. Biomed. J..

[19] Jimenez-Mateos E.M., Smith J., Nicke A., Engel T. (2019). Regulation of P2X7 receptor expression and function in the brain. Brain Res. Bull..

[20] Zhou L., Qi X., Potashkin J.A., Abdul-Karim F.W., Gorodeski G.I. (2008). MicroRNAs miR-186 and miR-150 Down-regulate Expression of the Pro-apoptotic Purinergic P2X7 Receptor by Activation of Instability Sites at the 3′-Untranslated Region of the Gene That Decrease Steady-state Levels of the Transcript. J. Biol. Chem..

[21] Oton-Gonzalez L., Rotondo J.C., Cerritelli L., Malagutti N., Lanzillotti C., Bononi I., Ciorba A., Bianchini C., Mazziotta C., De Mattei M. (2021). Association between oncogenic human papillomavirus type 16 and Killian polyp. Infect. Agent. Cancer.

[22] Rotondo J.C., Lanzillotti C., Mazziotta C., Tognon M., Martini F. (2021). Epigenetics of male infertility: The role of DNA methylation. Front. Cell Dev. Biol..

[23] Rotondo J.C., Mazziotta C., Lanzillotti C., Tognon M., Martini F. (2021). Epigenetic Dysregulations in Merkel Cell Polyomavirus-Driven Merkel Cell Carcinoma. Int. J. Mol. Sci..

[24] Lanzillotti C., De Mattei M., Mazziotta C., Taraballi F., Rotondo J.C., Tognon M., Martini F. (2021). Long Non-coding RNAs and MicroRNAs Interplay in Osteogenic Differentiation of Mesenchymal Stem Cells. Front. Cell Dev. Biol..

[25] Rotondo J.C., Giari L., Guerranti C., Tognon M., Castaldelli G., Fano E.A., Martini F. (2018). Environmental doses of perfluorooctanoic acid change the expression of genes in target tissues of common carp. Environ. Toxicol. Chem..

[26] Roger S., Jelassi B., Couillin I., Pelegrin P., Besson P., Jiang L.H. (2015). Understanding the roles of the P2X7 receptor in solid tumour progression and therapeutic perspectives. Biochim. Biophys. Acta—Biomembr..

[27] Li M., Luo S., Zhang Y., Jia L., Yang C., Peng X., Zhao R. (2020). Production, characterization, and application of a monoclonal antibody specific for the extracellular domain of human P2X7R. Appl. Microbiol. Biotechnol..

[28] Dalgarno R., Leduc-Pessah H., Pilapil A., Kwok C.H.T., Trang T. (2018). Intrathecal delivery of a palmitoylated peptide targeting Y382-384 within the P2X7 receptor alleviates neuropathic pain. Mol. Pain.

[29] Karasawa A., Michalski K., Mikhelzon P., Kawate T. (2017). The P2X7 receptor forms a dye-permeable pore independent of its intracellular domain but dependent on membrane lipid composition. eLife.

[30] Gonnord P., Delarasse C., Auger R., Benihoud K., Prigent M., Cuif M.H., Lamaze C., Kanellopoulos J.M. (2009). Palmitoylation of the P2X7 receptor, an ATP-gated channel, controls its expression and association with lipid rafts. FASEB J..

[31] Wickert L.E., Blanchette J.B., Waldschmidt N.V., Bertics P.J., Denu J.M., Denlinger L.C., Lenertz L.Y. (2013). The C-Terminus of Human Nucleotide Receptor P2X7 Is Critical for Receptor Oligomerization and N-Linked Glycosylation. PLoS ONE.

[32] Martínez-cuesta M.Á., Blanch-ruiz M.A., Ortega-luna R., Sánchez-lópez A., Álvarez Á., Karasawa A., Kawate T. (2020). Structural and Functional Basis for Understanding the Biological Significance of P2X7 Receptor. Int. J. Mol. Sci..

[33] Wiley J.S., Dao-Ung L.P., Li C., Shemon A.N., Gu B.J., Smart M.L., Fuller S.J., Barden J.A., Petrou S., Sluyter R. (2003). An Ile-568 to Asn Polymorphism Prevents Normal Trafficking and Function of the Human P2X7 Receptor. J. Biol. Chem..

[34] Bradley H.J., Liu X., Collins V., Owide J., Goli G.R., Smith M., Surprenant A., White S.J., Jiang L.H. (2010). Identification of an intracellular microdomain of the P2X7 receptor that is crucial in basolateral membrane targeting in epithelial cells. FEBS Lett..

[35] Smart M.L., Gu B., Panchal R.G., Wiley J., Cromer B., Williams D.A., Petrou S. (2003). P2X7 Receptor Cell Surface Expression and Cytolytic Pore Formation Are Regulated by a Distal C-terminal Region. J. Biol. Chem..

[36] Costa-Junior H.M., Vieira F.S., Coutinho-Silva R. (2011). C terminus of the P2X7 receptor: Treasure hunting. Purinergic Signal..

[37] Liu Y., Xiao Y., Li Z. (2011). P2X7 receptor positively regulates MyD88-dependent NF-κB activation. Cytokine.

[38] Jiang L.H., Caseley E.A., Muench S.P., Roger S. (2021). Structural basis for the functional properties of the P2X7 receptor for extracellular ATP. Purinergic Signal..

[39] Orioli E., De Marchi E., Giuliani A.L., Adinolfi E. (2017). P2X7 Receptor Orchestrates Multiple Signalling Pathways Triggering Inflammation, Autophagy and Metabolic/Trophic Responses. Curr. Med. Chem..

[40] Pelegrin P., Surprenant A. (2007). Pannexin-1 Couples to Maitotoxin- and Nigericin-induced Interleukin-1β Release through a Dye Uptake-independent Pathway. J. Biol. Chem..

[41] Baroja-Mazo A., Barberà-Cremades M., Pelegrín P. (2013). The participation of plasma membrane hemichannels to purinergic signaling. Biochim. Biophys. Acta—Biomembr..

[42] Di Virgilio F., Giuliani A.L., Vultaggio-Poma V., Falzoni S., Sarti A.C. (2018). Non-nucleotide agonists triggering P2X7 receptor activation and pore formation. Front. Pharmacol..

[43] North R.A. (2002). Molecular physiology of P2X receptors. Physiol. Rev..

[44] Bergamin L.S., Capece M., Salaro E., Sarti A.C., Falzoni S., Pereira M.S.L., De Bastiani M.A., Scholl J.N., Battastini A.M.O., Di Virgilio F. (2019). Role of the P2X7 receptor in in vitro and in vivo glioma tumor growth. Oncotarget.

[45] Roger S., Mei Z.Z., Baldwin J.M., Dong L., Bradley H., Baldwin S.A., Surprenant A., Jiang L.H. (2010). Single nucleotide polymorphisms that were identified in affective mood disorders affect ATP-activated P2X7 receptor functions. J. Psychiatr. Res..

[46] Young C.N.J., Górecki D.C. (2018). P2RX7 purinoceptor as a therapeutic target-The second coming?. Front. Chem..

[47] Young C.N.J., Chira N., Róg J., Al-Khalidi R., Benard M., Galas L., Chan P., Vaudry D., Zabłocki K., Górecki D.C. (2018). Sustained activation of P2X7 induces MMP-2-evoked cleavage and functional purinoceptor inhibition. J. Mol. Cell Biol..

[48] Wang Z., Ren W., Zhao F., Han Y., Liu C., Jia K. (2020). Curcumin amends Ca^2+^ dysregulation in microglia by suppressing the activation of P2X7 receptor. Mol. Cell. Biochem..

[49] Furini F., Giuliani A.L., Parlati M.E., Govoni M., Di Virgilio F., Bortoluzzi A. (2019). P2X7 receptor expression in patients with serositis related to systemic lupus erythematosus. Front. Pharmacol..

[50] Burnstock G., Knight G.E. (2018). The potential of P2X7 receptors as a therapeutic target, including inflammation and tumour progression. Purinergic Signal..

[51] Petrou S., Ugur M., Drummond R.M., Singer J.J., Walsh J.V. (1997). P2X7 purinoceptor expression in Xenopus oocytes is not sufficient to produce a pore-forming P2Z-like phenotype. FEBS Lett..

[52] Browne L.E., Compan V., Bragg L., North R.A. (2013). P2X7 Receptor Channels Allow Direct Permeation of Nanometer-Sized Dyes. J. Neurosci..

[53] Di Virgilio F., Jiang L.H., Roger S., Falzoni S., Sarti A.C., Vultaggio-Poma V., Chiozzi P., Adinolfi E. (2019). Structure, function and techniques of investigation of the P2X7 receptor (P2X7R) in mammalian cells. Methods Enzymol..

[54] Rossi L., Salvestrini V., Ferrari D., Di Virgilio F., Lemoli R.M. (2012). The sixth sense: Hematopoietic stem cells detect danger through purinergic signaling. Blood.

[55] Jiang L.H., Hao Y., Mousawi F., Peng H., Yang X. (2017). Expression of P2 Purinergic Receptors in Mesenchymal Stem Cells and Their Roles in Extracellular Nucleotide Regulation of Cell Functions. J. Cell. Physiol..

[56] Agrawal A., Gartland A. (2015). P2x7 receptors: Role in bone cell formation and function. J. Mol. Endocrinol..

[57] Fan X., Ma W., Zhang Y., Zhang L. (2020). P2X7 Receptor (P2X7R) of microglia mediates neuroinflammation by regulating (NOD)-like receptor protein 3 (NLRP3) inflammasome-dependent inflammation after spinal cord injury. Med. Sci. Monit..

[58] Kopp R., Krautloher A., Ramírez-Fernández A., Nicke A. (2019). P2X7 Interactions and Signaling—Making Head or Tail of It. Front. Mol. Neurosci..

[59] Giannuzzo A., Pedersen S.F., Novak I. (2015). The P2X7 receptor regulates cell survival, migration and invasion of pancreatic ductal adenocarcinoma cells. Mol. Cancer.

[60] Di Virgilio F., Ferrari D., Adinolfi E. (2009). P2X7: A growth-promoting receptor—Implications for cancer. Purinergic Signal..

[61] Gilbert S., Oliphant C., Hassan S., Peille A., Bronsert P., Falzoni S., Di Virgilio F., McNulty S., Lara R. (2019). ATP in the tumour microenvironment drives expression of nfP2X 7, a key mediator of cancer cell survival. Oncogene.

[62] Zhang Y., Ding J., Wang L. (2019). The role of P2X7 receptor in prognosis and metastasis of colorectal cancer. Adv. Med. Sci..

[63] Calik I., Calik M., Turken G., Ozercan I.H. (2020). A promising independent prognostic biomarker in colorectal cancer: P2X7 receptor. Int. J. Clin. Exp. Pathol..

[64] Li Q., Zhu X., Song W., Peng X., Zhao R. (2020). The P2X7 purinergic receptor: A potential therapeutic target for lung cancer. J. Cancer Res. Clin. Oncol..

[65] Maxová H., Bacáková L., Lisá V., Novotná J., Tomásová H., Vízek M., Herget J. (2010). Production of proteolytic enzymes in mast cells, fibroblasts, vascular smooth muscle and endothelial cells cultivated under normoxic or hypoxic conditions. Physiol. Res..

[66] Adinolfi E., Raffaghello L., Giuliani A.L., Cavazzini L., Capece M., Chiozzi P., Bianchi G., Kroemer G., Pistoia V., Di Virgilio F. (2012). Expression of P2X7 receptor increases in vivo tumor growth. Cancer Res..

[67] Tafani M., Schito L., Pellegrini L., Villanova L., Marfe G., Anwar T., Rosa R., Indelicato M., Fini M., Pucci B. (2011). Hypoxia-increased RAGE and P2X7R expression regulates tumor cell invasion through phosphorylation of Erk1/2 and Akt and nuclear translocation of NF-κB. Carcinogenesis.

[68] Zhang Y., Jiang M., Cui B.W., Jin C.H., Wu Y.L., Shang Y., Yang H.X., Wu M., Liu J., Qiao C.Y. (2020). P2X7 receptor-targeted regulation by tetrahydroxystilbene glucoside in alcoholic hepatosteatosis: A new strategy towards macrophage–hepatocyte crosstalk. Br. J. Pharmacol..

[69] Souza C.O., Santoro G.F., Figliuolo V.R., Nanini H.F., De Souza H.S.P., Castelo-Branco M.T.L., Abalo A.A., Paiva M.M., Coutinho C.M.L.M., Coutinho-Silva R. (2012). Extracellular ATP induces cell death in human intestinal epithelial cells. Biochim. Biophys. Acta—Gen. Subj..

[70] Hattori F., Ohshima Y., Seki S., Tsukimoto M., Sato M., Takenouchi T., Suzuki A., Takai E., Kitani H., Harada H. (2012). Feasibility study of B16 melanoma therapy using oxidized ATP to target purinergic receptor P2X7. Eur. J. Pharmacol..

[71] Amoroso F., Capece M., Rotondo A., Cangelosi D., Ferracin M., Franceschini A., Raffaghello L., Pistoia V., Varesio L., Adinolfi E. (2015). The P2X7 receptor is a key modulator of the PI3K/GSK3β/VEGF signaling network: Evidence in experimental neuroblastoma. Oncogene.

[72] Gu B.J., Wiley J.S. (2006). Rapid ATP-induced release of matrix metalloproteinase 9 is mediated by the P2X7 receptor. Blood.

[73] Xia J., Yu X., Tang L., Li G., He T. (2015). P2X7 receptor stimulates breast cancer cell invasion and migration via the AKT pathway. Oncol. Rep..

[74] Jelassi B., Chantme A., Alcaraz-Pérez F., Baroja-Mazo A., Cayuela M.L., Pelegrin P., Surprenant A., Roger S. (2011). P2X 7 receptor activation enhances SK3 channels- and cystein cathepsin-dependent cancer cells invasiveness. Oncogene.

[75] Bekes E.M., Schweighofer B., Kupriyanova T.A., Zajac E., Ardi V.C., Quigley J.P., Deryugina E.I. (2011). Tumor-recruited neutrophils and neutrophil TIMP-free MMP-9 regulate coordinately the levels of tumor angiogenesis and efficiency of malignant cell intravasation. Am. J. Pathol..

[76] Murphy N., Lynch M.A. (2012). Activation of the P2X7 receptor induces migration of glial cells by inducing cathepsin B degradation of tissue inhibitor of metalloproteinase 1. J. Neurochem..

[77] Arnaud-Sampaio V.F., Rabelo I.L.A., Ulrich H., Lameu C. (2020). The P2X7 Receptor in the Maintenance of Cancer Stem Cells, Chemoresistance and Metastasis. Stem Cell Rev. Rep..

[78] Schneider G., Glaser T., Lameu C., Abdelbaset-Ismail A., Sellers Z.P., Moniuszko M., Ulrich H., Ratajczak M.Z. (2015). Extracellular nucleotides as novel, underappreciated pro-metastatic factors that stimulate purinergic signaling in human lung cancer cells. Mol. Cancer.

[79] Takai E., Tsukimoto M., Harada H., Kojima S. (2014). Autocrine signaling via release of ATP and activation of P2X7 receptor influences motile activity of human lung cancer cells. Purinergic Signal..

[80] Jelassi B., Anchelin M., Chamouton J., Cayuela M.L., Clarysse L., Li J., Goré J., Jiang L.H., Roger S. (2013). Anthraquinone emodin inhibits human cancer cell invasiveness by antagonizing P2X7 receptors. Carcinogenesis.

[81] Amoroso F., Salaro E., Falzoni S., Chiozzi P., Giuliani A.L., Cavallesco G., Maniscalco P., Puozzo A., Bononi I., Martini F. (2016). P2X7 targeting inhibits growth of human mesothelioma. Oncotarget.

[82] Gómez-Villafuertes R., García-Huerta P., Díaz-Hernández J.I., Miras-Portugal M.T. (2015). PI3K/Akt signaling pathway triggers P2X7 receptor expression as a pro-survival factor of neuroblastoma cells under limiting growth conditions. Sci. Rep..

[83] Amoroso F., Falzoni S., Adinolfi E., Ferrari D., Di Virgilio F. (2012). The P2X7 receptor is a key modulator of aerobic glycolysis. Cell Death Dis..

[84] Liu M., Huang X., Tian Y., Yan X., Wang F., Chen J., Zhang Q., Zhang Q., Yuan X. (2020). Phosphorylated GSK-3β protects stress-induced apoptosis of myoblasts via the PI3K/Akt signaling pathway. Mol. Med. Rep..

[85] Kunnimalaiyaan S., Schwartz V.K., Jackson I.A., Clark Gamblin T., Kunnimalaiyaan M. (2018). Antiproliferative and apoptotic effect of LY2090314, a GSK-3 inhibitor, in neuroblastoma in vitro. BMC Cancer.

[86] Qin L.X. (2012). Inflammatory Immune Responses in Tumor Microenvironment and Metastasis of Hepatocellular Carcinoma. Cancer Microenviron..

[87] Pellegatti P., Raffaghello L., Bianchi G., Piccardi F., Pistoia V., Di Virgilio F. (2008). Increased Level of Extracellular ATP at Tumor Sites: In Vivo Imaging with Plasma Membrane Luciferase. PLoS ONE.

[88] Savio L.E.B., Mello P.d.A., da Silva C.G., Coutinho-Silva R. (2018). The P2X7 receptor in inflammatory diseases: Angel or demon?. Front. Pharmacol..

[89] Burnstock G. (2016). P2X ion channel receptors and inflammation. Purinergic Signal..

[90] Lopez-Castejon G., Brough D. (2011). Understanding the mechanism of IL-1β secretion. Cytokine Growth Factor Rev..

[91] Carta S., Rubartelli R.L. (2013). Different Members of the IL-1 Family Come Out in Different Ways: DAMPs vs. Cytokines?. Front. Immunol..

[92] Scarpellino G., Genova T., Munaron L. (2019). Purinergic P2X7 Receptor: A Cation Channel Sensitive to Tumor Microenvironment. Recent Pat. Anticancer. Drug Discov..

[93] Di Virgilio F., Dal Ben D., Sarti A.C., Giuliani A.L., Falzoni S. (2017). The P2X7 Receptor in Infection and Inflammation. Immunity.

[94] Killeen M.E., Ferris L., Kupetsky E.A., Falo L., Mathers A.R. (2013). Signaling through Purinergic Receptors for ATP Induces Human Cutaneous Innate and Adaptive Th17 Responses: Implications in the Pathogenesis of Psoriasis. J. Immunol..

[95] Eberhardt N., Bergero G., Mazzocco Mariotta Y.L., Aoki M.P. (2022). Purinergic modulation of the immune response to infections. Purinergic Signal..

[96] Soare A.Y., Freeman T.L., Min A.K., Malik H.S., Osota E.O., Swartz T.H. (2021). P2RX7 at the Host-Pathogen Interface of Infectious Diseases. Microbiol. Mol. Biol. Rev..

[97] Zhang C., He H., Wang L., Zhang N., Huang H., Xiong Q., Yan Y., Wu N., Ren H., Han H. (2017). Virus-Triggered ATP Release Limits Viral Replication through Facilitating IFN-β Production in a P2X7-Dependent Manner. J. Immunol..

[98] Corrêa G., Marques da Silva C., de Abreu Moreira-Souza A.C., Vommaro R.C., Coutinho-Silva R. (2010). Activation of the P2X(7) receptor triggers the elimination of Toxoplasma gondii tachyzoites from infected macrophages. Microbes Infect..

[99] Lee B.H., Hwang D.M., Palaniyar N., Grinstein S., Philpott D.J., Hu J. (2012). Activation of P2X7 Receptor by ATP Plays an Important Role in Regulating Inflammatory Responses during Acute Viral Infection. PLoS ONE.

[100] Paoletti A., Raza S.Q., Voisin L., Law F., Pipoli da Fonseca J., Caillet M., Kroemer G., Perfettini J.L. (2012). Multifaceted roles of purinergic receptors in viral infection. Microbes Infect..

[101] Pacheco P.A.F., Faria R.X., Ferreira L.G.B., Paixão I.C.N.P. (2014). Putative roles of purinergic signaling in human immunodeficiency virus-1 infection. Biol. Direct.

[102] Barat C., Gilbert C., Imbeault M., Tremblay M.J. (2008). Extracellular ATP reduces HIV-1 transfer from immature dendritic cells to CD4+ T lymphocytes. Retrovirology.

[103] Taylor J.M., Han Z. (2010). Purinergic Receptor Functionality Is Necessary for Infection of Human Hepatocytes by Hepatitis Delta Virus and Hepatitis B Virus. PLoS ONE.

[104] Ribeiro D.E., Oliveira-Giacomelli Á., Glaser T., Arnaud-Sampaio V.F., Andrejew R., Dieckmann L., Baranova J., Lameu C., Ratajczak M.Z., Ulrich H. (2020). Hyperactivation of P2X7 receptors as a culprit of COVID-19 neuropathology. Mol. Psychiatry.

[105] Franciosi M.L.M., Lima M.D.M., Schetinger M.R.C., Cardoso A.M. (2021). Possible role of purinergic signaling in COVID-19. Mol. Cell. Biochem..

[106] Rotondo J.C., Martini F., Maritati M., Mazziotta C., Di Mauro G., Lanzillotti C., Barp N., Gallerani A., Tognon M., Contini C. (2021). SARS-CoV-2 Infection: New Molecular, Phylogenetic, and Pathogenetic Insights. Efficacy of Current Vaccines and the Potential Risk of Variants. Viruses.

[107] Contini C., Rotondo J.C., Magagnoli F., Maritati M., Seraceni S., Graziano A., Poggi A., Capucci R., Vesce F., Tognon M. (2018). Investigation on silent bacterial infections in specimens from pregnant women affected by spontaneous miscarriage. J. Cell. Physiol..

[108] Coutinho-Silva R., Perfettini J.L., Persechini P.M., Dautry-Varsat A., Ojcius D.M. (2001). Modulation of P2Z/P2X7 receptor activity in macrophages infected with Chlamydia psittaci. Am. J. Physiol.—Cell Physiol..

[109] Abdul-Sater A.A., Saïd-Sadier N., Padilla E.V., Ojcius D.M. (2010). Chlamydial infection of monocytes stimulates IL-1β secretion through activation of the NLRP3 inflammasome. Microbes Infect..

[110] Santiago-Carvalho I., de Almeida-Santos G., Bomfim C.C.B., Souza P.C.D., Melo B.M.S.D., Amaral E.P., Cione M.V.P., Lasunskaia E., Hirata M.H., Alves-Filho J.C.F. (2021). P2x7 Receptor Signaling Blockade Reduces Lung Inflammation and Necrosis During Severe Experimental Tuberculosis. Front. Cell. Infect. Microbiol..

[111] Csóka B., Németh Z.H., Töro G., Idzko M., Zech A., Koscsó B., Spolarics Z., Antonioli L., Cseri K., Erdélyi K. (2015). Extracellular ATP protects against sepsis through macrophage P2X7 purinergic receptors by enhancing intracellular bacterial killing. FASEB J..

[112] Savio L.E.B., de Andrade Mello P., Figliuolo V.R., de Avelar Almeida T.F., Santana P.T., Oliveira S.D.S., Silva C.L.M., Feldbrügge L., Csizmadia E., Minshall R.D. (2017). CD39 limits P2X7 receptor inflammatory signaling and attenuates sepsis-induced liver injury. J. Hepatol..

[113] Van De Veerdonk F.L., Joosten L.A.B., Devesa I., Héctor M.M.M., Kanneganti T.D., Dinarello C.A., Van Der Meer J.W.M., Gow N.A.R., Kullberg B.J., Netea M.G. (2009). Bypassing Pathogen-Induced Inflammasome Activation for the Regulation of Interleukin-1β Production by the Fungal Pathogen Candida albicans. J. Infect. Dis..

[114] Feriotti C., de Araújo E.F., Loures F.V., da Costa T.A., Galdino N.A.D.L., Zamboni D.S., Calich V.L.G. (2017). NOD-like receptor P3 inflammasome controls protective Th1/Th17 immunity against pulmonary paracoccidioidomycosis. Front. Immunol..

[115] Snelgrove R.J., Gregory L.G., Peiró T., Akthar S., Campbell G.A., Walker S.A., Lloyd C.M. (2014). Alternaria-derived serine protease activity drives IL-33–mediated asthma exacerbations. J. Allergy Clin. Immunol..

[116] Quan J.H., Huang R., Wang Z., Huang S., Choi I.W., Zhou Y., Lee Y.H., Chu J.Q. (2018). P2X7 receptor mediates NLRP3-dependent IL-1β secretion and parasite proliferation in Toxoplasma gondii-infected human small intestinal epithelial cells. Parasites Vectors.

[117] Chaves S.P., Torres-Santos E.C., Marques C., Figliuolo V.R., Persechini P.M., Coutinho-Silva R., Rossi-Bergmann B. (2009). Modulation of P2X7 purinergic receptor in macrophages by Leishmania amazonensis and its role in parasite elimination. Microbes Infect..

[118] Oliveira S.D.A.d.S., Coutinho-Silva R., Silva C.L.M. (2012). Endothelial P2X7 receptors’ expression is reduced by schistosomiasis. Purinergic Signal..

[119] Oliveira S.D.A.S., Nanini H.F., Savio L.E.B., Waghabi M.C., Silva C.L.M., Coutinho-Silva R. (2014). Macrophage P2X7 Receptor Function is Reduced during Schistosomiasis: Putative Role of TGF-β 1. Mediators Inflamm..

[120] Cruz L.N., Wu Y., Ulrich H., Craig A.G., Garcia C.R.S. (2016). Tumor necrosis factor reduces Plasmodium falciparum growth and activates calcium signaling in human malaria parasites. Biochim. Biophys. Acta—Gen. Subj..

[121] Levano-Garcia J., Dluzewski A.R., Markus R.P., Garcia C.R.S. (2010). Purinergic signalling is involved in the malaria parasite Plasmodium falciparum invasion to red blood cells. Purinergic Signal..

[122] Hamarsheh S., Zeiser R. (2020). NLRP3 Inflammasome Activation in Cancer: A Double-Edged Sword. Front. Immunol..

[123] Makarenkova H.P., Shah S.B., Shestopalov V.I. (2018). The two faces of pannexins: New roles in inflammation and repair. J. Inflamm. Res..

[124] Hung S.C., Choi C.H., Said-Sadier N., Johnson L., Atanasova K.R., Sellami H., Yilmaz Ö., Ojcius D.M. (2013). P2X4 Assembles with P2X7 and Pannexin-1 in Gingival Epithelial Cells and Modulates ATP-induced Reactive Oxygen Species Production and Inflammasome Activation. PLoS ONE.

[125] Draganov D., Gopalakrishna-Pillai S., Chen Y.R., Zuckerman N., Moeller S., Wang C., Ann D., Lee P.P. (2015). Modulation of P2X4/P2X7/Pannexin-1 sensitivity to extracellular ATP via Ivermectin induces a non-apoptotic and inflammatory form of cancer cell death. Sci. Rep..

[126] Miao E.A., Rajan J.V., Aderem A. (2011). Caspase-1-induced pyroptotic cell death. Immunol. Rev..

[127] Lin L., Huang S., Zhu Z., Han J., Wang Z., Huang W., Huang Z. (2018). P2X7 receptor regulates EMMPRIN and MMP-9 expression through AMPK/MAPK signaling in PMA-induced macrophages. Mol. Med. Rep..

[128] Corazza M., Oton-Gonzalez L., Scuderi V., Rotondo J.C., Lanzillotti C., Di Mauro G., Tognon M., Martini F., Borghi A. (2020). Tissue cytokine/chemokine profile in vulvar lichen sclerosus: An observational study on keratinocyte and fibroblast cultures. J. Dermatol. Sci..

[129] Siebers K., Fink B., Zakrzewicz A., Agné A., Richter K., Konzok S., Hecker A., Zukunft S., Küllmar M., Klein J. (2018). Alpha-1 antitrypsin inhibits ATP-mediated release of interleukin-1β via CD36 and nicotinic acetylcholine receptors. Front. Immunol..

[130] Rotondo J.C., Oton-Gonzalez L., Selvatici R., Rizzo P., Pavasini R., Campo G.C., Lanzillotti C., Mazziotta C., De Mattei M., Tognon M. (2020). SERPINA1 Gene Promoter Is Differentially Methylated in Peripheral Blood Mononuclear Cells of Pregnant Women. Front. Cell Dev. Biol..

[131] Rotondo J.C., Aquila G., Oton-Gonzalez L., Selvatici R., Rizzo P., De Mattei M., Pavasini R., Tognon M., Campo G.C., Martini F. (2021). Methylation of SERPINA1 gene promoter may predict chronic obstructive pulmonary disease in patients affected by acute coronary syndrome. Clin. Epigenet..

[132] Lotfi R., Steppe L., Hang R., Rojewski M., Massold M., Jahrsdörfer B., Schrezenmeier H. (2018). ATP promotes immunosuppressive capacities of mesenchymal stromal cells by enhancing the expression of indoleamine dioxygenase. Immun. Inflamm. Dis..

[133] Di Virgilio F., Sarti A.C., Falzoni S., De Marchi E., Adinolfi E. (2018). Extracellular ATP and P2 purinergic signalling in the tumour microenvironment. Nat. Rev. Cancer.

[134] Zhang Y., Cheng H., Li W., Wu H., Yang Y. (2019). Highly-expressed P2X7 receptor promotes growth and metastasis of human HOS/MNNG osteosarcoma cells via PI3K/Akt/GSK3β/β-catenin and mTOR/HIF1α/VEGF signaling. Int. J. Cancer.

[135] Ji Z., Xie Y., Guan Y., Zhang Y., Cho K.S., Ji M., You Y. (2018). Involvement of P2X7 Receptor in Proliferation and Migration of Human Glioma Cells. Biomed Res. Int..

[136] Vijayan D., Young A., Teng M.W.L., Smyth M.J. (2017). Targeting immunosuppressive adenosine in cancer. Nat. Rev. Cancer.

[137] Antonioli L., Blandizzi C., Pacher P., Haskó G. (2013). Immunity, inflammation and cancer: A leading role for adenosine. Nat. Rev. Cancer.

[138] Li M., Toombes G.E.S., Silberberg S.D., Swartz K.J. (2015). Physical basis of apparent pore dilation of ATP-activated P2X receptor channels. Nat. Neurosci..

[139] Acuna C., Capelli C., Coddou C., Escobar A., Imarai M., Yohana L., Lopex X., Rios M., Lopez M. (2014). WO2013023319A1—In Vitro Method for Modifying the Depletion Profile of Treg Cells Present in a Total Splenocyte Population of a Biological Sample by Means of the Isolation, Culturing and Exposure Thereof to an Atp and Polymixin b Medium. U.S. Patent.

[140] Feng L.L., Cai Y.Q., Zhu M.C., Xing L.J., Wang X. (2020). The yin and yang functions of extracellular ATP and adenosine in tumor immunity. Cancer Cell Int..

[141] Avanzato D., Genova T., Fiorio Pla A., Bernardini M., Bianco S., Bussolati B., Mancardi D., Giraudo E., Maione F., Cassoni P. (2016). Activation of P2X7 and P2Y11 purinergic receptors inhibits migration and normalizes tumor-derived endothelial cells via cAMP signaling. Sci. Rep..

[142] Barden J.A. (2014). Non-Functional P2X7: A Novel and Ubiquitous Target in Human Cancer. J. Clin. Cell. Immunol..

[143] Stokes L., Bidula S., Bibič L., Allum E. (2020). To Inhibit or Enhance? Is There a Benefit to Positive Allosteric Modulation of P2X Receptors?. Front. Pharmacol..

[144] Donnelly-Roberts D.L., Namovic M.T., Han P., Jarvis M.F. (2009). Mammalian P2X7 receptor pharmacology: Comparison of recombinant mouse, rat and human P2X7 receptors. Br. J. Pharmacol..

[145] Solini A., Santini E., Chimenti D., Chiozzi P., Pratesi F., Cuccato S., Falzoni S., Lupi R., Ferrannini E., Pugliese G. (2007). Multiple P2X receptors are involved in the modulation of apoptosis in human mesangial cells: Evidence for a role of P2X4. Am. J. Physiol.—Ren. Physiol..

[146] Adriouch S., Hubert S., Pechberty S., Koch-Nolte F., Haag F., Seman M. (2007). NAD + Released during Inflammation Participates in T Cell Homeostasis by Inducing ART2-Mediated Death of Naive T Cells In Vivo. J. Immunol..

[147] Sanz J.M., Chiozzi P., Ferrari D., Colaianna M., Idzko M., Falzoni S., Fellin R., Trabace L., Di Virgilio F. (2009). Activation of Microglia by Amyloid β Requires P2X 7 Receptor Expression. J. Immunol..

[148] Elssner A., Duncan M., Gavrilin M., Wewers M.D. (2004). A Novel P2X 7 Receptor Activator, the Human Cathelicidin-Derived Peptide LL37, Induces IL-1β Processing and Release. J. Immunol..

[149] Ferrari D., Pizzirani C., Gulinelli S., Callegari G., Chiozzi P., Idzko M., Panther E., Di Virgilio F. (2007). Modulation of P2X 7 receptor functions by polymyxin B: Crucial role of the hydrophobic tail of the antibiotic molecule. Br. J. Pharmacol..

[150] Greve A.S., Skals M., Fagerberg S.K., Tonnus W., Ellermann-Eriksen S., Evans R.J., Linkermann A., Praetorius H.A. (2017). P2X1, P2X4, and P2X7 Receptor Knock Out Mice Expose Differential Outcome of Sepsis Induced by α-Haemolysin Producing Escherichia coli. Front. Cell. Infect. Microbiol..

[151] Allsopp R.C., Dayl S., Schmid R., Evans R.J. (2017). Unique residues in the ATP gated human P2X7 receptor define a novel allosteric binding pocket for the selective antagonist AZ10606120. Sci. Rep..

[152] Allsopp R.C., Dayl S., Dayel A.B., Schmid R., Evans R.J. (2018). Mapping the allosteric action of antagonists A740003 and A438079 reveals a role for the left flipper in ligand sensitivity at P2X7 receptorss. Mol. Pharmacol..

[153] Bartlett R., Stokes L., Sluyter R. (2014). The p2x7 receptor channel: Recent developments and the use of p2x7 antagonists in models of disease. Pharmacol. Rev..

[154] Karoutzou O., Kwak S.H., Lee S.D., Martínez-Falguera D., Sureda F.X., Vázquez S., Kim Y.C., Barniol-Xicota M. (2018). Towards a Novel Class of Multitarget-Directed Ligands: Dual P2X7–NMDA Receptor Antagonists. Molecules.

[155] Chhabra P., Linden J., Lobo P., Douglas Okusa M., Lewis Brayman K. (2012). The Immunosuppressive Role of Adenosine A2A Receptors in Ischemia Reperfusion Injury and Islet Transplantation. Curr. Diabetes Rev..

[156] Kan L.K., Seneviratne S., Drummond K.J., Williams D.A., O’Brien T.J., Monif M. (2020). P2X7 receptor antagonism inhibits tumour growth in human high-grade gliomas. Purinergic Signal..

[157] Tognon M., Tagliapietra A., Magagnoli F., Mazziotta C., Oton-Gonzalez L., Lanzillotti C., Vesce F., Contini C., Rotondo J.C., Martini F. (2020). Investigation on Spontaneous Abortion and Human Papillomavirus Infection. Vaccines.

[158] Tognon M., Luppi M., Corallini A., Taronna A., Barozzi P., Rotondo J.C., Comar M., Casali M.V., Bovenzi M., D’Agostino A. (2015). Immunologic evidence of a strong association between non-Hodgkin lymphoma and simian virus 40. Cancer.

[159] Mazziotta C., Pellielo G., Tognon M., Martini F., Rotondo J.C. (2021). Significantly low levels of IgG antibodies against oncogenic Merkel cell polyomavirus in sera from females affected by spontaneous abortion. Front. Microbiol..

[160] Rotondo J.C., Mazzoni E., Bononi I., Tognon M.G., Martini F. (2019). Association Between Simian Virus 40 and Human Tumors. Front. Oncol..

[161] Mazziotta C., Rotondo J.C., Lanzillotti C., Campione G., Martini F., Tognon M. (2021). Cancer biology and molecular genetics of A3 adenosine receptor. Oncogene.

[162] Vincenzi F., Rotondo J.C., Pasquini S., Di Virgilio F., Varani K., Tognon M. (2021). A3 Adenosine and P2X7 Purinergic Receptors as New Targets for an Innovative Pharmacological Therapy of Malignant Pleural Mesothelioma. Front. Oncol..

[163] Drill M., Powell K.L., Kan L.K., Jones N.C., O’Brien T.J., Hamilton J.A., Monif M. (2020). Inhibition of purinergic P2X receptor 7 (P2X7R) decreases granulocyte-macrophage colony-stimulating factor (GM-CSF) expression in U251 glioblastoma cells. Sci. Rep..

[164] Giannuzzo A., Saccomano M., Napp J., Ellegaard M., Alves F., Novak I. (2016). Targeting of the P2X7 receptor in pancreatic cancer and stellate cells. Int. J. Cancer.

[165] Zhang W.J., Luo C., Huang C., Pu F.Q., Zhu J.F., Zhu Z. (2021). ming PI3K/Akt/GSK-3β signal pathway is involved in P2X7 receptor-induced proliferation and EMT of colorectal cancer cells. Eur. J. Pharmacol..

[166] Talreja O., Kerndt C.C., Cassagnol M. (2021). Simvastatin. xPharm Compr. Pharmacol. Ref..

[167] Stock T.C., Bloom B.J., Wei N., Ishaq S., Park W., Wang X., Gupta P., Mebus C.A. (2012). Efficacy and Safety of CE-224,535, an Antagonist of P2X7 Receptor, in Treatment of Patients with Rheumatoid Arthritis Inadequately Controlled by Methotrexate. J. Rheumatol..

[168] McInnes I.B., Schett G. (2007). Cytokines in the pathogenesis of rheumatoid arthritis. Nat. Rev. Immunol..

[169] Drill M., Jones N.C., Hunn M., O’Brien T.J., Monif M. (2021). Antagonism of the ATP-gated P2X7 receptor: A potential therapeutic strategy for cancer. Purinergic Signal..

[170] Deng Y., Zhou M., Zhao X., Xue X., Liao L., Wang J., Li Y. (2022). Immune response studies based on P2X7 receptors: A Mini-Review. Curr. Pharm. Des..

[171] Sheng D., Hattori M. (2022). Recent progress in the structural biology of P2X receptors. Proteins Struct. Funct. Bioinform..

